# Thyroid hormone action controls multiple components of cell junctions at the ventricular zone in the newborn rat brain

**DOI:** 10.3389/fendo.2023.1090081

**Published:** 2023-02-10

**Authors:** Katherine L. O’Shaughnessy, Benjamin D. McMichael, Aubrey L. Sasser, Kiersten S. Bell, Cal Riutta, Jermaine L. Ford, Tammy E. Stoker, Rachel D. Grindstaff, Arun R. Pandiri, Mary E. Gilbert

**Affiliations:** ^1^ United States Environmental Protection Agency, Public Health Integrated Toxicology Division, Center for Public Health and Environmental Assessment, Research Triangle Park, NC, United States; ^2^ Oak Ridge Institute for Science Education, Oak Ridge, TN, United States; ^3^ Chemical Characterization and Exposure Division, Center for Computational Toxicology and Exposure, United States Environmental Protection Agency, Research Triangle Park, NC, United States; ^4^ Comparative and Molecular Pathogenesis Branch, Division of Translational Toxicology, National Institute of Environmental Health Sciences, Research Triangle Park, NC, United States

**Keywords:** thyroid hormone action, hypothyroidism, brain development, ventricular zone, cell migration, radial glia, cell adhesion, cell junctions

## Abstract

Thyroid hormone (TH) action controls brain development in a spatiotemporal manner. Previously, we demonstrated that perinatal hypothyroidism led to formation of a periventricular heterotopia in developing rats. This heterotopia occurs in the posterior telencephalon, and its formation was preceded by loss of radial glia cell polarity. As radial glia mediate cell migration and originate in a progenitor cell niche called the ventricular zone (VZ), we hypothesized that TH action may control cell signaling in this region. Here we addressed this hypothesis by employing laser capture microdissection and RNA-Seq to evaluate the VZ during a known period of TH sensitivity. Pregnant rats were exposed to a low dose of propylthiouracil (PTU, 0.0003%) through the drinking water during pregnancy and lactation. Dam and pup THs were quantified postnatally and RNA-Seq of the VZ performed in neonates. The PTU exposure resulted in a modest increase in maternal thyroid stimulating hormone and reduced thyroxine (T4). Exposed neonates exhibited hypothyroidism and T4 and triiodothyronine (T3) were also reduced in the telencephalon. RNA-Seq identified 358 differentially expressed genes in microdissected VZ cells of hypothyroid neonates as compared to controls (q-values ≤0.05). Pathway analyses showed processes like maintenance of the extracellular matrix and cytoskeleton, cell adhesion, and cell migration were significantly affected by hypothyroidism. Immunofluorescence also demonstrated that collagen IV, F-actin, radial glia, and adhesion proteins were reduced in the VZ. Immunohistochemistry of integrin αvβ3 and isoforms of both thyroid receptors (TRα/TRβ) showed highly overlapping expression patterns, including enrichment in the VZ. Taken together, our results show that TH action targets multiple components of cell junctions in the VZ, and this may be mediated by both genomic and nongenomic mechanisms. Surprisingly, this work also suggests that the blood-brain and blood-cerebrospinal fluid barriers may also be affected in hypothyroid newborns.

## 1 Introduction

Thyroid hormone (TH) action controls multiple developmental pathways in the brain (reviewed in 1). These thyroid-dependent processes all exhibit striking spatiotemporal activity, with subcompartments of the brain exhibiting differing sensitivities to THs, which also varies with developmental time (1, 2).

Highlighting these spatiotemporal dynamics, we previously reported that a transient maternal exposure to the propylthiouracil (PTU) resulted in the formation of a periventricular heterotopia in the developing rat brain (3, 4). This malformation is not only inducible by anti-thyroid pharmaceuticals, but also by environmental thyroid disrupting chemicals in multiple strains of rats (3–9). The heterotopia is predominantly comprised of ectopic neurons and can be detected in 100% of pups following a 5-day perinatal exposure (gestational day 19 – postnatal day 2, GD19 – PN2) (4). In addition to a clear temporal susceptibility, we also identified a spatial sensitivity to TH action. The heterotopia reproducibly occurs in the posterior telencephalon, directly medial to the lateral ventricular epithelium as this region extends into the corpus callosum (3, 5–8). Given these data, we hypothesized that the developing ventricular epithelium is acutely sensitive to reduced TH action during the perinatal period in the rat. We postulated that this may be due in part to its anatomical location. The most luminal cells come in direct contact the cerebrospinal fluid (CSF), one source of brain THs (10). The ventricular epithelium also possesses an enriched vascular network, and as THs are also actively transported across the blood-brain barrier (BBB), the microvasculature represents a second source of brain T4/T3 (4, 11). Thus, the cells of the ventricular epithelium reside at the intersection of TH transport.

In addition to being a potential target of TH action, the neonatal ventricular epithelium is also one of two stem cell niches that supports neurogenesis. The most apical proliferative population is the ventricular zone (VZ), and houses multipotent stem cells, namely radial glia (12–14). Importantly, while radial glia are progenitors, they also act as migratory scaffolding to mediate the travel of neuroblasts into regions like the cortex (14). The next most basal cell layer is called the subventricular zone (SVZ), which also possesses undifferentiated cells including intermediate progenitors (14–16). Unlike the SVZ, which supports neurogenesis throughout life, the VZ is only present during embryonic and early postnatal development in rodents (13). The VZ appears as the pseudostratified ventricular epithelium due to the cell bodies of numerous radial glia (13). By approximately 2-weeks of age, the pseudostratified epithelium remodels to simple columnar as radial glia cells differentiate, leaving the SVZ with only limited proliferative capacity (13). These dynamics between VZ/SVZ transition during the postnatal period, in addition to the VZ’s anatomical location, may also contribute to TH susceptibility.

Here we investigated the hypothesis that the posterior VZ is a target of TH action during the neonatal period in rats. We exposed pregnant animals to a low dose of propylthiouracil (PTU) beginning in early pregnancy. Using laser capture microdissection (LCM), we isolated cells of the posterior VZ in pups during a known period of hormone sensitivity (PN2) (4). We then employed RNA-Sequencing (RNA-Seq) and pathway analyses to investigate the molecular pathways disturbed in affected neonates. Next, we performed immunohistochemistry to garner further support of our RNA-Seq findings, which includes mediators of TH action.

## 2 Materials and methods

### 2.1 Animal husbandry and exposure

All experiments were conducted with prior approval from the United States Environmental Protection Agency’s (US EPA’s) Institutional Animal Care and Usage Committee and were carried out in an Association for Assessment and Accreditation of Laboratory Animal Care approved facility. N=16 timed pregnant Long Evans rats were purchased from Charles River (Morrisville, NC) and delivered on gestational day (GD) 2; sperm positive was considered GD0 and pup birth postnatal day 0 (PN0). Dams were single housed in polycarbonate cages, maintained a 12:12 light cycle and offered chow (Purina 5008) and deionized water ad libitum. Animals were weight ranked and then randomly allocated to two treatment groups. N=8 dams were exposed to 3 ppm (0.0003%) 6-propyl-2-thiouracil (PTU, purity ≥98%, Sigma) dissolved in deionized drinking water; N=8 control dams were administered vehicle only (deionized drinking water). The maternal exposure was initiated on GD6 and continued through PN14. This PTU exposure was not expected to induce overt toxicity in dams, but was sufficient to induce a periventricular heterotopia in their offspring (3). N=8 controls and N=7 PTU exposed dams gave birth.

### 2.2 Serum thyroid hormone quantification

To evaluate serum total thyroxine (T4), triiodothyronine (T3), and thyroid stimulating hormone (TSH), pups on PN2 and dams on PN14 were euthanized by rapid decapitation and trunk blood collected in serum separator tubes with clot activating gel (BD Vacutainer). Serum hormones were quantified from N=8 vehicle control and N=7 PTU exposed dams and litters. Pup blood was pooled from two littermates, taking one male and one female from each litter when possible. Previous work has shown that PTU exposure similarly reduces serum T4 and T3 in both sexes of rats (17, 18). Blood samples were allowed to clot on ice for at least 30 minutes before centrifugation at 1300 x g (4° C). Serum was then aliquoted in sterile, nonstick tubes and stored at -80°C until analysis. Pups were analyzed on PN2 due to our previous work demonstrating that TH dysfunction at this stage is associated with periventricular heterotopia development (4). The dams were euthanized at the conclusion of this animal exposure on PN14; other data collected from this animal cohort will be published elsewhere. For further information on dam serum TH profiles over the perinatal period following a 3 ppm PTU exposure, see (3).

Serum T4 and T3 were analyzed by liquid chromatography with tandem mass spectrometry using an AB Sciex (Framingham, Massachusetts) Exion AC UHPLC-Qtrap 6500+ Linear Ion Trap LC/MS/MS system as previously described (19). The lower limit of quantitation (LLOQ) for each analyte was set to the concentration of the lowest calibration standard that gave an acceptable ion ratio, and acceptable recovery of ±30% of the spike amount; the lower limit of quantification (LLOQ) for both T4/T3 were 0.1 ng/ml (100 pg/ml). Each sample batch consisted of a method blank, a laboratory control sample (blank spike), and a continuing calibration verification sample prepared in the solvent. Serum TSH was analyzed on using a Milliplex Rat Thyroid Hormone Magnetic Bead Panel (Millipore Sigma, RTHYMAG-30K-01) according to the manufacturer’s protocol. The curve fit R^2^ was >0.99 and the sum of residuals was -0.004. Unpaired, two-tailed Welch’s t-tests with an α=0.05 were performed for each measured analyte using GraphPad Prism 9.1.2 (GraphPad Software, San Diego, CA).

### 2.3 Brain hormone quantification

Following rapid decapitation, the brain from one female pup per litter was extracted from the skull and the telencephalon (forebrain) isolated in sterile 0.01 M phosphate buffered saline (PBS). Tissue was blotted to remove excess buffer, weighed, placed in sterile tubes, and frozen in liquid nitrogen. All samples were stored at -80°C until analysis. THs were isolated by solid phase extraction and analyzed by LC/MS/MS as previously described (19) for N=5 control and N=5 PTU exposed samples. The LLOQ for total T4 and reverse T3 (rT3) were 0.01 ng/g and total T3 0.05 ng/g. Unpaired, two-tailed Welch’s t-tests with an α=0.05 were performed for each TH measured using GraphPad Prism 9.1.2 (GraphPad Software, San Diego, CA). Previous work has shown no differences in heterotopia incidence or severity between the sexes, suggesting that sex not a significant variable in determination of this phenotype (3). Hence, only female brain hormones were assessed.

### 2.4 Laser capture microdissection

One male pup was selected from each of N=7 control and N=6 PTU exposed litters on PN2 for laser capture microdissection (LCM) and sequencing. Pups were administered an overdose of Euthasol^®^ and transcardiac perfusion performed with 30% sucrose in sterile 0.01 M PBS pH 7.4. Following sucrose perfusion, the brain was immediately dissected from the skull, embedded in Tissue Freezing Medium™ (Fisher Scientific, 15-183-13), and frozen on a slurry of dry ice and isopentane. The brains were cryosectioned coronally at 10 µm thickness and collected directly onto MicroDissect polyethylene terephthalate membrane single frame slides (ASEE, FS-LMD-M-50r); slides were placed on dry ice immediately after section pickup and stored -80°C. Immediately before microdissection slides were stained with 2% cresyl violet in 75% ethanol, dehydrated, and cleared. Laser capture of the posterior ventricular epithelium was then performed using a MMI CellCut LCM System (Molecular Machines and Industries). The epithelium as identified by its pseudostratified appearance, consistent with reports that this morphology represents the VZ (13). Isolated cells were then collected using 0.5 ml MicroDissect stick-cap tubes (ASEE, ST-LMD-M-500). The ventricular epithelium from both hemispheres of ~20 sections was collected from each animal.

### 2.5 RNA isolation

RNA was isolated using Qiagen’s RNeasy^®^ Micro kit (Qiagen, 74004). Immediately after cells from each slide were collected as described, 10 µl of RLT lysis buffer was added directly on to the cap for 5 minutes. RNA was isolated from lysed cells according to the manufacturer’s protocol, including on-column DNA digestion. RNA concentrations were determined using Qubit (RNA HS, Q32852) and quality using Agilent Bioanalyzer (Nano, 5067-1511). The average RNA concentration across biological replicates was 15 ng/µl and RNA Integrity Number was (RIN) 5.3. While this indicates some RNA degradation, slide control cells (microdissected CA1/CA3 of the hippocampus) revealed consistently intact RNA with RIN ≥8 across all biological replicates (**Supplementary Figure 1**). Therefore, we attributed the lower RINs of our microdissected VZ to the long, narrow shape of this region, which likely resulted in an increased amount of burned cellular debris relative to intact cells following microdissection.

### 2.6 Library preparation and RNA-Sequencing (RNA-Seq)

Total RNA-Sequencing libraries were prepared using the SMARTer^®^ Stranded Total RNA-Seq Kit v3 - Pico Input Mammalian kit (Takara, 634485) according to the manufacturer’s protocol. This included sample barcoding and rRNA depletion. Library quantities were evaluated using Qubit (dsDNA, Q32851) and library size and quality evaluated using Agilent Bioanalyzer (DNA HS, 5067-4626). Libraries were pooled and sequenced on two lanes of Illumina HiSeq 4000 and paired end sequenced to an average depth of 58 million reads/sample.

### 2.7 Identifying differentially expressed genes (DEGs)

Samples were demultiplexed and trimmed using Trimmomatic (20). Downstream processing of RNA-Seq data was performed in Partek Flow Bioinformatics Software (Chesterfield, MO). Samples were aligned to the *Rattus norvegicus* reference genome (mRatBN7.2/rn7) using STAR 2.7.8a. Counts were quantified using the Partek Expectation/Maximization (E/M) model and the rat RefSeq annotation (GCF_015227675.2) and normalized using median ratio. Differential expression was identified by comparing N=6 PTU exposed samples to N=7 controls using DESeq2 (21), and multiple testing corrected using the Benjamini–Hochberg step-up procedure to control the False Discovery Rate (FDR). DEGs were identified by an FDR (q-value) ≤0.05. A volcano plot was generated using the ggplot2 R package (22), and a heatmap of normalized count values using the Pheatmap (23) and Viridis R packages (24).

### 2.8 Gene ontology and pathway analyses

For preliminary investigation of the RNA-Seq data, all differentially expressed genes (DEGs) with a q-value ≤0.05 were analyzed using Gene Ontology (GO) in STRING (25). Results within the sub-ontologies of Cellular Components and Biological Function are reported here, with an adjusted p-value (q) ≤0.05 considered significant. Next, significant DEGs (q ≤ 0.05) were analyzed in Ingenuity Pathway Analysis (IPA) (Qiagen, Hilden, Germany) and used to generate predicted canonical and disease and function pathway analyses between control and PTU exposure.

### 2.9 Immunohistochemistry and imaging

A subset of PN2 pups of both sexes were euthanized for immunohistochemistry. Pups were administered an overdose of Euthasol^®^ and perfused with sterile 0.01 M PBS pH 7.4, and then 4% paraformaldehyde. Brains were isolated and cryoprotected in 30% sucrose in PBS before embedding in Tissue Freezing Medium™ (Fisher Scientific, 15-183-13). Blocks were frozen on a slurry of dry ice and isopentane. The brains were cryosectioned coronally at 35 µm and collected in PBS for free-floating immunohistochemistry (IHC). All primary and secondary antibody combinations can be found in **Supplementary Table 1**. For fluorescent IHC, nonspecific binding was blocked in a mixture of 10% horse and goat serum in PBS with 0.1% triton-X for 2 hours. Primary antibodies were then diluted in block buffer and incubated overnight at 4°C. The sections were then washed in PBS and incubated with appropriate Alexa Fluor antibodies and counterstained with DAPI. For visualization of filamentous actin (F-actin), sections were incubated in 0.5% triton for 15 minutes before incubation in Alexa Fluor 647 phalloidin according to manufacturer’s protocol (Abcam, ab176759). Fluorescent sections were then mounted on SuperFrost Plus (Fisher Scientific, 12-550-15) slides and coverslipped using ProLong Diamond (Thermo Fisher Scientific, P36965). Imaging was performed using a Nikon A1 laser scanning confocal microscope fitted with an Eclipse Ti inverted microscope base and a T-P2 Nikon polarizer slider. Control sections were imaged first and PTU exposed animals analyzed using the same parameters (see laser line information in **Supplementary Table 1**). For calorimetric detection background staining was reduced by incubating in 0.3% hydrogen peroxide diluted in methanol for 30 minutes. Nonspecific binding was reduced in block as stated above, and the signal amplified by avidin-biotin complex (ABC kit, Vector PK-4001 and PK-4002); the color reaction was developed with 3,3′-Diaminobenzidine (Sigma, D8001). Slides were then mounted, dehydrated, cleared, and cover slipped before imaging using an Aperio slide scanner (Leica). For all immunohistochemistry experiments at least N=3 control and N=3 PTU exposed pups were analyzed, and all images are representative of repeated findings.

### 2.10 Figure preparation

Microscopy images were prepared in Adobe Photoshop and figures assembled in Adobe Illustrator, with the same settings applied across control and PTU exposed images.

## 3 Results

### 3.1 PTU exposure perturbed the maternal thyroid axis

**Figure 1:**
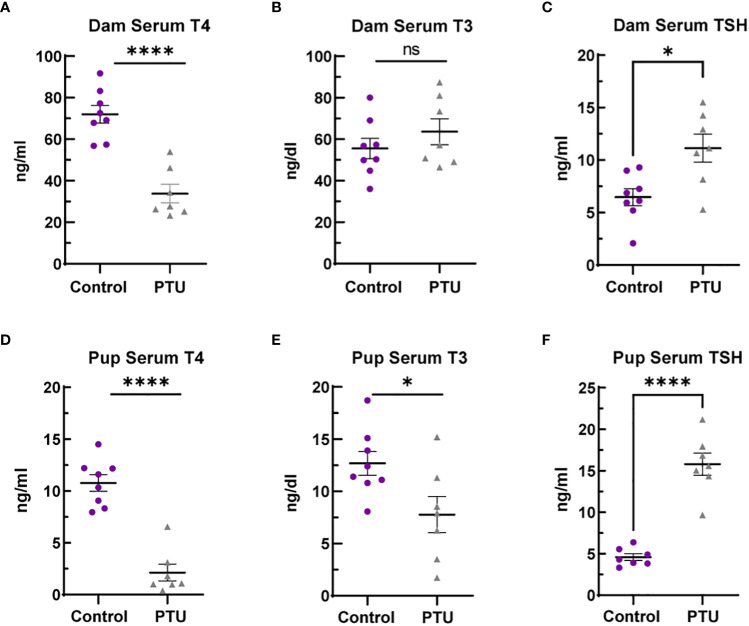
Serum thyroid hormones in dams and pups. **(A)** Dam serum T4 is reduced in dams on PN14, whereas **(B)** total T3 was not significantly different. **(C)** TSH was increased by 72% in PTU exposed dams on PN14. **(D)** Pup serum T4 and **(E)** T3 were reduced in pups on PN2. **(F)** TSH was increased in PN2 neonates. In all presented data, N=8 control and N=7 PTU exposed dams or litters were assayed. For all pup data, each biological replicate represents serum pooled from multiple littermates of mixed sex. The data were then analyzed by t-test and each graph shows the value of each biological replicate and the SEM; ****p ≤ 0.0001, *p ≤ 0.05, ns, not significant.

Following the drinking water exposure beginning on GD6, PTU significantly reduced dam T4 by 53% as compared to vehicle controls on PN14 (**Figure 1A**, p<0.0001). In contrast, there was no significant change in serum T3 (**Figure 1B**, p=0.33). Serum TSH was increased by 72% (**Figure 1C**, p=0.01). Together, the T4/T3/TSH results shows that PTU exposed dams exhibited thyroid axis perturbation.

### 3.2 Neonatal pups exhibited hypothyroidism and decreased brain T4/T3

**Figure 2:**
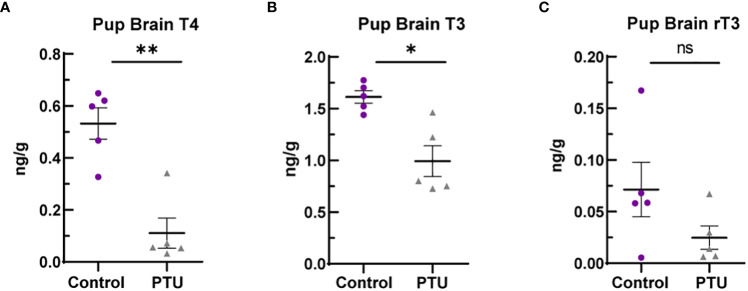
Telencephalon thyroid hormones in PN2 pups. **(A)** Brain T4 was significantly reduced on PN2, as was **(B)** T3. **(C)** Reverse T3 (rT3) was reduced in PTU exposed animals when comparing group means, but this was not statistically significant. In all presented data, N=8 control and N=7 PTU exposed female pups were assayed (1 pup/litter). The data were then analyzed by t-test and each graph shows the value of each biological replicate and the SEM; **p ≤ 0.001, *p ≤ 0.05, ns, not significant.

On PN2, a known day of hormone susceptibility (4), serum T4 was reduced in neonates by 80% (p<0.0001) (**Figure 1D**) and serum T3 by 39% (p=0.03) (**Figure 1E**). Serum TSH was increased by 243% (p<0.0001) (**Figure 1F**). In the telencephalon, T4 was significantly reduced by 80% (p=0.001) and T3 by 38% (p=0.02) (**Figures 2A, B**). Reverse T3 (rT3) was not significantly different following PTU exposure in the neonatal telencephalon (p=0.16) but showed more biological variability than T4/T3 (**Figure 2C**). Together, these serum and brain data show that the pups exhibited overt hypothyroidism and decreased brain tissue T4/T3 concentrations.

### 3.3 TH action regulates gene expression at the neonatal ventricular epithelium

**Figure 3:**
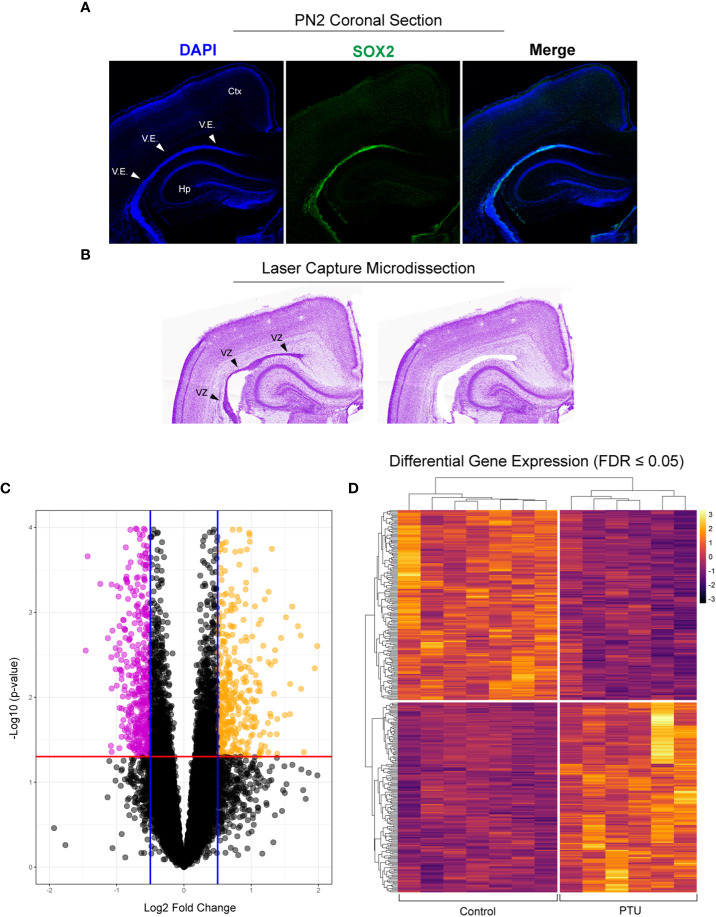
Laser capture microdissection and RNA-Seq of the neonatal ventricular zone (VZ). **(A)** Fluorescent immunostaining in control animals demonstrates that the ventricular epithelium (V.E.) of the posterior telencephalon is highly enriched for SOX2, demonstrating that this is the VZ (ventricular zone, progenitor cell niche) on postnatal day 2 (PN2). **(B)** Example of laser capture microdissection of the VZ. **(C)** Volcano plot of resulting RNA-Seq data obtained from microdissected VZ from PN2 male pups, with downregulated (pink) and upregulated (yellow) genes highlighted (q ≤ 0.05). **(D)** These differentially expressed genes (DEGs) were then visualized by a heatmap, which shows clear differences between the control (euthyroid) and PTU exposed (hypothyroid) neonates. Each column of the heatmap represents a biological replicate (N=7 control and N, 6 PTU exposed pups).

To confirm that the posterior ventricular epithelium is the VZ, we performed SRY-Box 2 (SOX2) immunofluorescence. SOX2 is expressed in multipotent neural stem cells, and is enriched in the VZ (26). Results show that in the posterior telencephalon, strong SOX2 expression was observed in the PN2 ventricular epithelium, which appears pseudostratified when observed by DAPI nuclear staining (**Figure 3A**). Following laser capture microdissection of this region and RNA-Seq (**Figure 3B**), 358 DEGs with FDR ≤0.05 were identified in male pups (172 downregulated, 186 upregulated) (**Figures 3B–D**, see **Supplementary File 1** for full results). Preliminary examination of the dataset revealed a subset of DEGs related to extracellular matrix composition. This included 4 collagen genes with a q<0.05 (*Col8a2*, *Col2a1*, *Col12a1*, *Col4a6*), and 3 approaching statistical significance (q ≤ 0.07, *Col9a3*, *Col25a1*, *Col4a5*) (**Table 1**, which includes full gene names). All of these collagen transcripts were downregulated except for *Col25a1*. Several other genes encoding extracellular matrix proteins were also significantly downregulated, including a glypican, syndecan, and dystroglyan (*Glp4*, *Sdc2*, *Dag1*). Another subset of DEGs were also related to cytoskeleton formation and/or dynamics. Four genes related to actin or tubulin formation were upregulated (*Actg1*, *Tbcb*, *Tpgs2*, *Tubb2b*, and *Tubb4a*, **Table 1**); three unconventional myosins, which bind F-actin, were all downregulated (*Myo1d*, *Myo1e*, *Myo7a*). A third subgroup of DEGs related to cell adhesion and/or cell junctions were downregulated, including *Cldn1* and *Jam3* (**Table 1**). Further, genes related to Wnt/Planar cell polarity (*Vangl1*, *Vangl2*), hedgehog signaling (*Gli2*, *Gli3*), and bone morphogenetic protein (BMP) signaling (*Rgma*, *Bmp1*, *Bmper*) were also all downregulated in PTU exposed VZ cells (**Table 1**). Downregulation of *Spred1* was also detected, a gene we previously implicated in heterotopia development (4). Surprisingly, only 3 genes were identified as known mediators of TH transport and/or action, including *Slc16a2*, *Tshr*, and *Thrsp*, all of which were downregulated (**Table 1**); no deiodinase enzymes (i.e., *Dio2*) or known T4/T3 receptors (i.e., *Thra*, *Thrb*) were differentially expressed in the VZ (**Supplementary File 1**). Genes known to respond to lowered brain T4/T3, like *Klf9* and *Hr*, were also not amongst the DEGs (**Supplementary File 1**). We acknowledge that downregulation of *Slc16a2* (MCT8) was a counterintuitive finding. Given that our brain hormone measures show a significant reduction in T3, one would expect upregulation of *Slc16a2* to increase T3 availability at the VZ. While we do not understand why *Slc16a2* is downregulated, it is important to consider if this signal is originating from specific cell types, or is similar across all *Slc16a2* expressing cells. As the apical VZ abuts TH containing CSF and the VZ possesses an enriched BBB, there may be complex mechanisms of regulating TH economy in this region that we cannot discern by bulk RNA-Seq. The lack of transcriptional signal for deiodinases and thyroid receptors is less surprising, as previous gene expression studies of mild/moderate developmental hypothyroidism in the postnatal rat brain have reported similar data (4, 27, 28).

**Table 1:** Genes of interest from RNA-Seq dataset.

Gene Name	Ensembl ID	Description	p-value	q-value	Fold change
*Gpc4*	ENSRNOG00000002413	glypican 4	<0.001	0.00	-1.71
*Sdc2*	ENSRNOG00000004936	syndecan 2	<0.001	0.02	-1.42
*Frem2*	ENSRNOG00000021670	FRAS1 related extracellular matrix 2	<0.001	0.04	-1.35
*Dag1*	ENSRNOG00000019400	dystroglycan 1	<0.001	0.04	-1.24
*Col8a2*	ENSRNOG00000010841	collagen type VIII alpha 2 chain	<0.001	0.02	-1.80
*Col2a1*	ENSRNOG00000058560	collagen type II alpha 1 chain	<0.001	0.02	-1.93
*Col12a1*	ENSRNOG00000058470	collagen type XII alpha 1 chain	<0.001	0.04	-1.36
*Col4a6*	ENSRNOG00000056772	collagen type IV alpha 6 chain	<0.001	0.04	-1.45
*Col9a3*	ENSRNOG00000009531	collagen type IX alpha 3 chain	<0.01	0.06	-1.45
*Col25a1*	ENSRNOG00000050706	collagen type XXV alpha 1 chain	<0.01	0.06	2.45
*Col4a5*	ENSRNOG00000018951	collagen, type IV, alpha 5	<0.01	0.07	-1.37
*Tbcb*	ENSRNOG00000020781	tubulin folding cofactor B	<0.001	0.01	1.47
*Tpgs2*	ENSRNOG00000054118	tubulin polyglutamylase complex subunit 2	<0.001	0.02	1.38
*Tubb2b*	ENSRNOG00000017445	tubulin, beta 2B class Iib	<0.001	0.04	1.22
*Actg1*	ENSRNOG00000036701	actin, gamma 1	<0.001	0.02	1.21
*Myo1d*	ENSRNOG00000003276	myosin ID	<0.001	0.02	-1.61
*Myo1e*	ENSRNOG00000061928	myosin IE	<0.001	0.02	-1.39
*Myo7a*	ENSRNOG00000013641	myosin VIIA	<0.001	0.04	-1.55
*Ajap1*	ENSRNOG00000050137	adherens junctions associated protein 1	<0.001	0.02	2.71
*Jam3*	ENSRNOG00000009149	junctional adhesion molecule 3	<0.001	0.02	-1.29
*Celsr1*	ENSRNOG00000021285	Cadherin EGF LAG seven-pass receptor 1	<0.001	0.02	-1.37
*Cldn1*	ENSRNOG00000001926	claudin 1	<0.01	0.05	-1.81
*Hepacam*	ENSRNOG00000009219	hepatic and glial cell adhesion molecule	<0.01	0.05	-1.32
*Vangl1*	ENSRNOG00000016477	VANGL planar cell polarity protein 1	<0.001	0.02	-1.53
*Vangl2*	ENSRNOG00000004889	VANGL planar cell polarity protein 2	<0.001	0.02	-1.30
*Gli2*	ENSRNOT00000009963	GLI family zinc finger 2	<0.001	0.03	-1.34
*Gli3*	ENSRNOT00000019396	GLI family zinc finger 3	<0.001	0.05	-1.35
*Bmp1*	ENSRNOG00000010890	bone morphogenetic protein 1	<0.001	0.04	-1.35
*Bmper*	ENSRNOG00000015357	BMP-binding endothelial regulator	<0.01	0.05	-1.38
*Slc16a2*	ENSRNOG00000002832	monocarboxylic acid transporter 8	<0.001	0.02	-1.49
*Tshr*	ENSRNOG00000003972	thyroid stimulating hormone receptor	<0.001	0.02	-1.41
*Thrsp*	ENSRNOG00000012404	thyroid hormone responsive	<0.001	0.04	-1.42
*Spred1*	ENSRNOG00000070996	sprouty-related EVH1 domain containing 1	<0.001	0.02	-1.25

**Figure 4:**
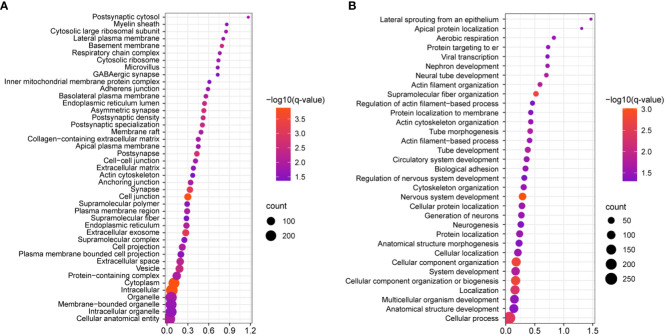
Statistically significant gene ontology (GO) analyses of the identified DEGs, as visualized by bubble plots. **(A)** Cell component GO reveals that the most statistically significant categories include intracellular, cytoplasm, and cell junction (q=0.00013 for each); this shows that most protein products DEGs will localize to these cellular compartments. **(B)** Biological processes analysis shows that cell component organization, nervous system development, cytoskeletal organization, and supramolecular fiber organization are amongst the most statistically significant processes (q≤0.0018), while more specific categories like cytoskeletal organization, biological adhesion, actin filament-based processes, were also identified. In both panels all categories listed are supported by q≤0.05.

**Figure 5:**
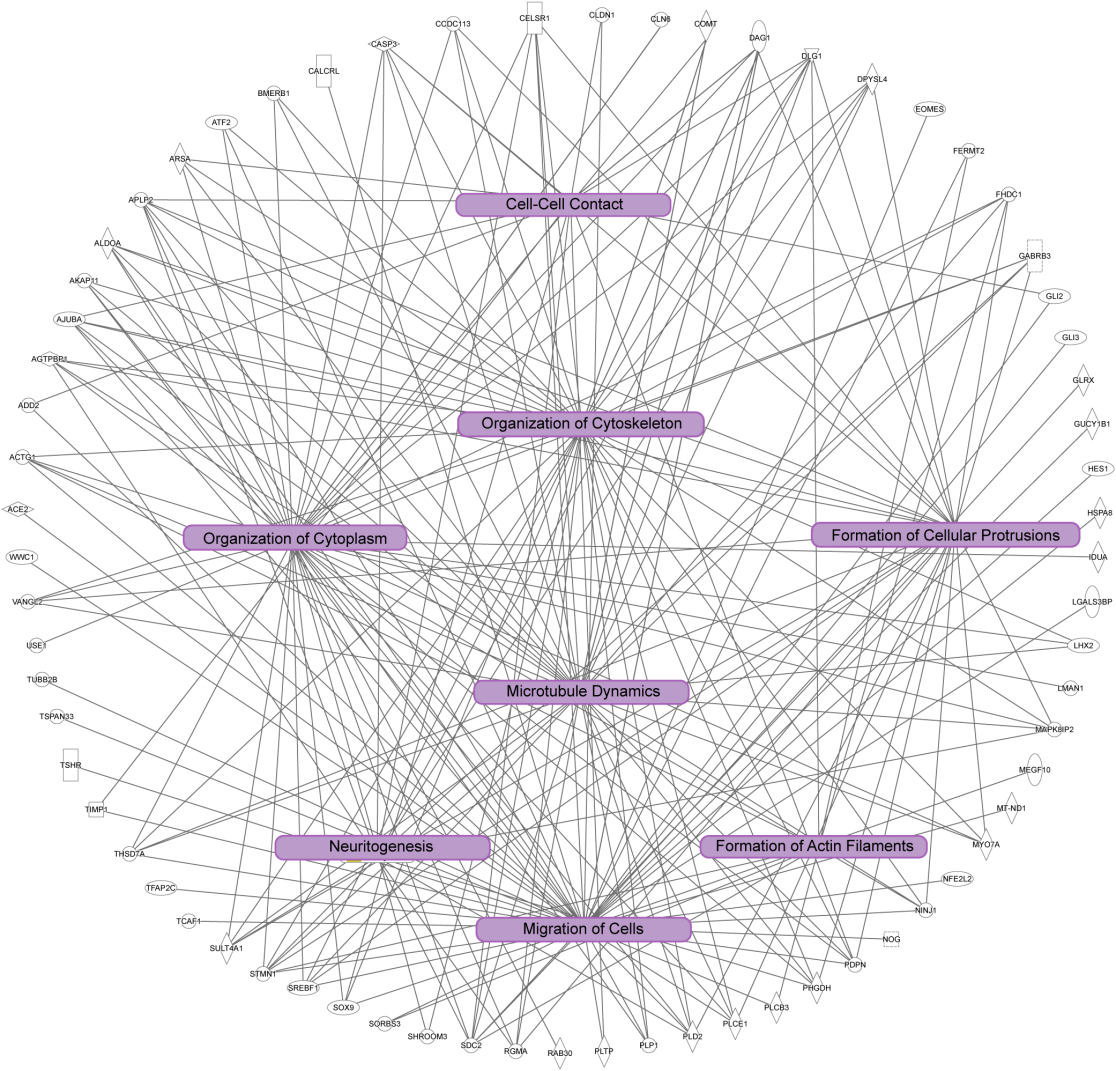
Ingenuity pathway analysis of significant cellular pathways. In this diagram unique genes identified are the outside of the circle, and connected to statistically significant processes (p<0.001). Note the overlap of the biological processes highlighted in purple boxes, which are correlated to dynamics of the cytoskeleton and cytoplasm, cell adhesion, cell morphology, and cell migration.

We next investigated the signaling pathways with overrepresentation amongst the DEGs. Gene Ontology (GO) Cellular Component analysis showed most gene products will localize intracellularly, within the cytoplasm, and at the cell junction (**Figure 4A** and **Supplementary File 2**). Among the most significant Biological Processes identified include nervous system development (q<0.001), cellular component organization (q=0.002), fiber organization (q=0.002), actin filament organization (q=0.02), lateral sprouting from an epithelium (q=.03), adhesion (q=0.03), and cytoskeletal organization (q=0.03) (**Figure 4B** and **Supplementary File 3**). A transcriptional signal for circulatory system development was also detected (q=0.03). Ingenuity Pathway Analysis showed similar results as GO Biological Processes. The top Molecular and Cellular Functions include Cellular Assembly and Organization (54 DEGs associated, p-values <0.001) as well as Cell-to-Cell Signaling and Interaction (36 DEGs, p-values <0.001). Between these two categories, there were 68 unique DEGs (**Figure 5**). Highly significant processes within this curated data include cell-cell contact, organization of cytoplasm, organization of cytoskeleton, formation of cellular protrusions, microtubule dynamics, neuritogenesis, formation of actin, and cell migration (**Figure 5**, all p-values <0.001). Ingenuity Pathway Analysis’s Canonical Pathways also revealed both different and overlapping signals in our DEGs. The most significant Canonical Pathways were oxidative phosphorylation, mitochondrial dysfunction, and protein kinase A signaling; cell-cell junction as well as endothelin-1 signaling were also significant in our data set (all p-values <0.001) (see **Supplementary File 4**).

### 3.4 Immunofluorescence demonstrates thyroid-dependent changes in cell junction components of the VZ

**Figure 6:**
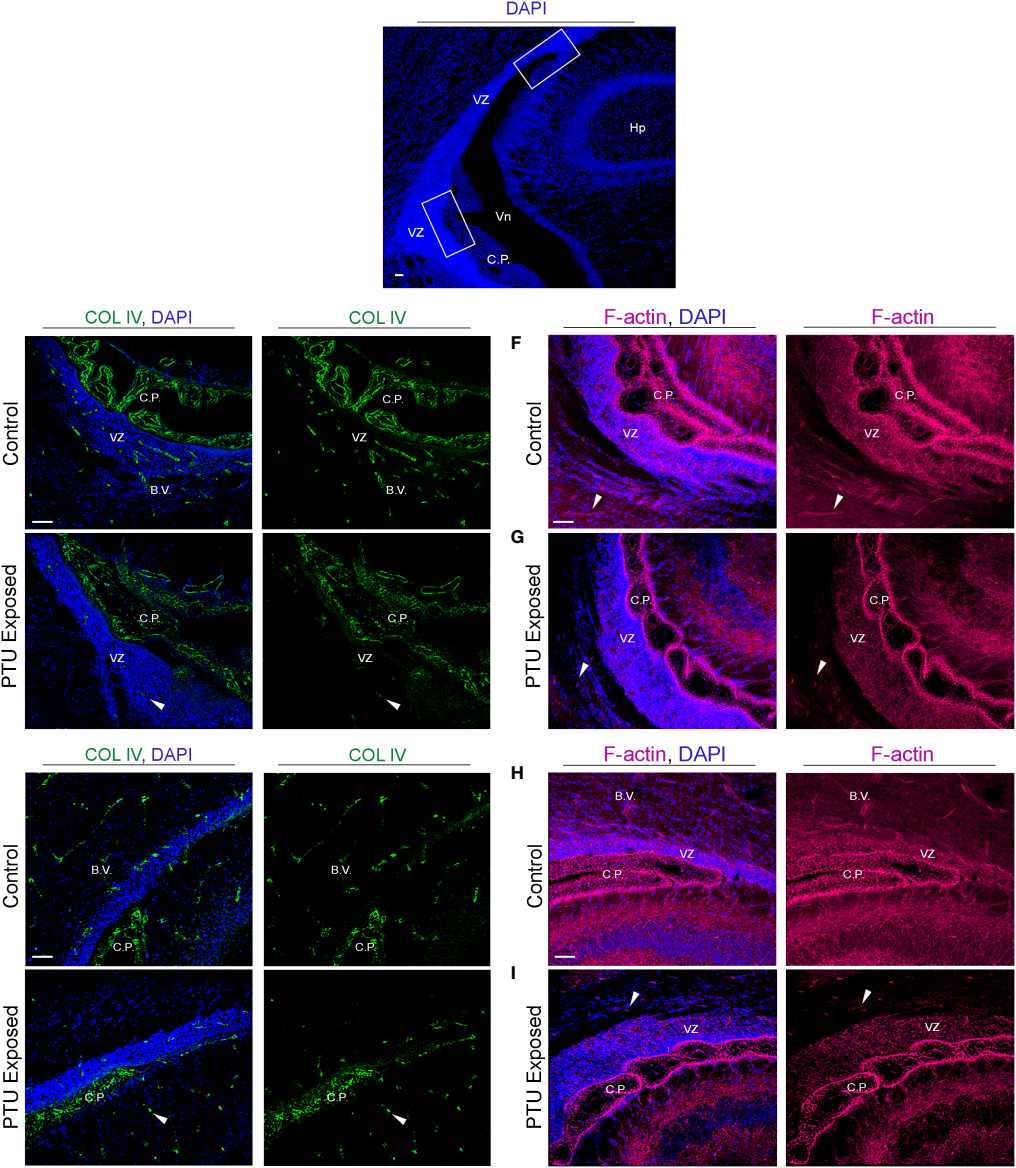
Developmental hypothyroidism affects collagen IV (COL IV) and filamentous actin (F-actin) in PN2 neonates **(A)** Representative regions of the posterior telencephalon. The white bonding boxes represent the inferior and superior portions of the VZ, and represent where the images were captured. Note that the choroid plexus (C.P., blood-cerebrospinal fluid barrier) is in proximity to the VZ and thus often imaged simultaneously. **(B)** Collagen IV (COL IV) is an extracellular matrix protein that is critical to basement membrane maintenance in cerebral endothelial and in some epithelial cells, and was implicated by RNA-Seq. In control pups, COL IV is enriched in blood vessels (B.V.) of the inferior VZ, as well as the vascular component of the choroid plexus. Less pronounced staining is also observed in the more luminal area of the VZ. **(C)** In PTU exposed pups, there was a clear disorganization of COL IV in the blood vessels, the choroid plexus, and in the inferior VZ parenchyma (see arrows). **(D)** COL IV is expressed in the blood vessels and choroid plexus of the superior VZ. **(E)** In PTU exposed pups COL IV also appears reduced in the superior VZ. **(F)** Visualization of F-actin by phalloidin shows strong signal throughout the brain, including in the blood vessels of the inferior VZ. F-actin was also highly enriched surrounding the nuclei of cells (blue DAPI staining) in the VZ, as expected. **(G)** In the PTU exposed animals, staining appeared globally reduced, which was especially notable in the parenchymal cells surrounding the VZ as well as in blood vessels (see arrows). **(H)** F-actin is highly expressed in the superior VZ and its associated blood vessels in control animals. **(I)** The PTU-associated changes in F-actin are apparent in the VZ and the surrounding parenchna. In all panels VZ, ventricular zone; C.P., choroid plexus; Hp, hippocampus; B.V., blood vessel; Vn, ventricle and scale bars are 50 µm.

**Figure 7:**
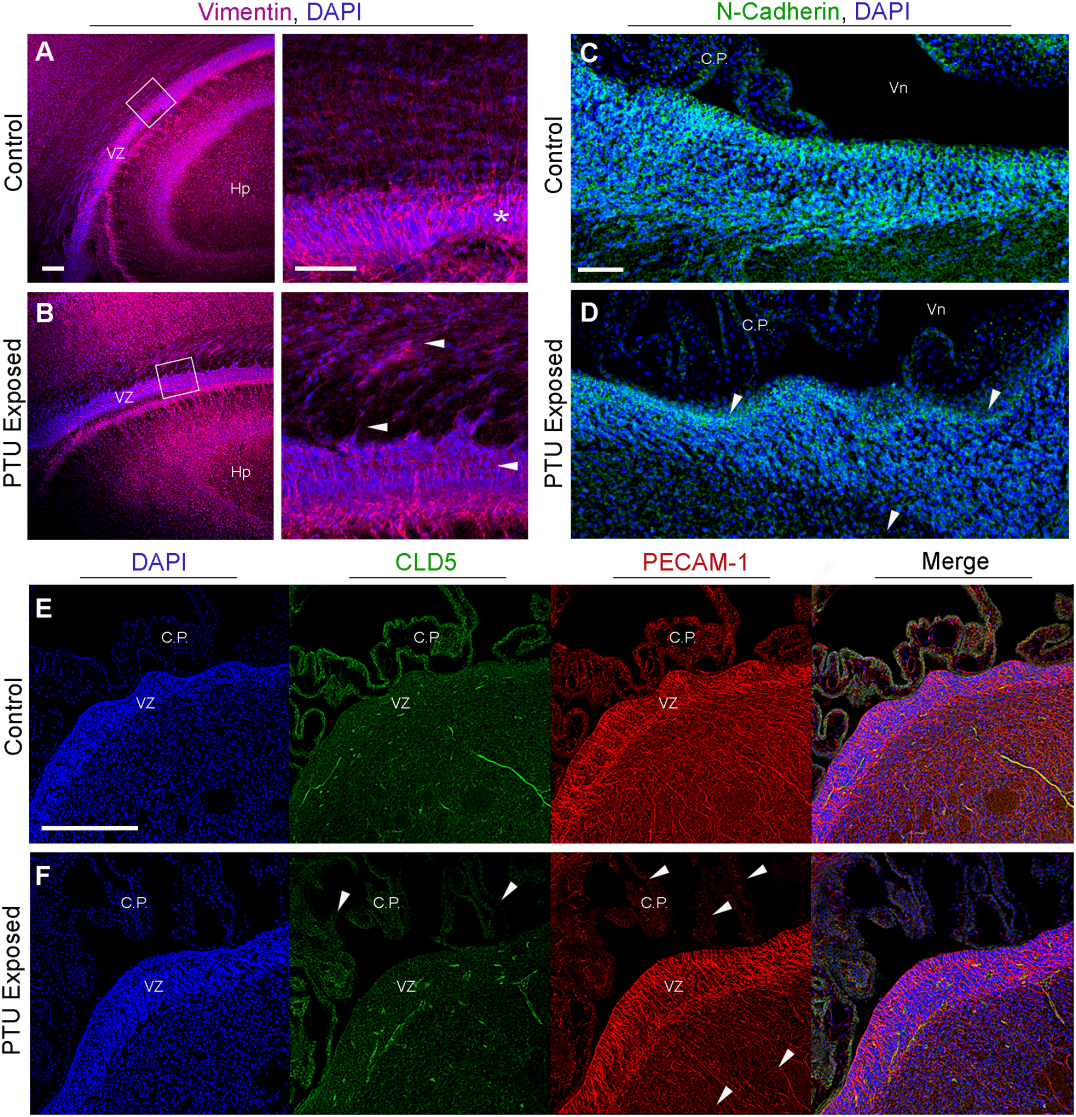
Radial glia cells and cell adhesion are affected in the newborn VZ. **(A)** Vimentin immunohistochemistry highlights radial glia cells. In control animals radial glia endfeet anchor these cells to the ventricular zone (VZ), as seen at high magnification (see asterisk). **(B)** In PTU exposed pups, there is a loss of the apicobasal polarity of radial glia, which is especially prominent at high magnification (see white arrows). Note that the high magnification image was rotated so that the VZ was viewed horizontally. **(C)** Visualization of adherens junctions by N-cadherin. N-cadherin expression is observed in the VZ, notably along the apical border which faces cerebrospinal fluid. **(D)** In PTU exposed pups on PN2 there is a disorganization of N-Cadherin, especailly along the apical border (see arrows). **(E)** Examination of tight junctions by claudin 5 (CLD5) and endothelial cell by PECAM-1. CLD5 is normally expressed in endothelial cells and is a critical compoenent of the blood-brain and blood-cerebrospinal fluid barriers. It is also expressed along the apical surface of the VZ, similar to N-Cadherin. **(F)** In hypothyroid (PTU exposed) neontates, there was a pronounced reduction and disorganization in CLD5 staining in the choroid plexus (see arrows). There was also subtle changes in CLD5 along the apical surface of the VZ. Interestingly, PECAM-1 was also affected in the choroid plexus, and at the more basal region of the VZ as it transitions to the SVZ (see arrows). In all panels VZ, ventricular zone; C.P., choroid plexus; Hp, hippocampus; B.V., blood vessel; Vn, ventricle, and scale bars are 50 µm.

To corroborate the RNA-Seq findings, we next performed immunofluorescence to visualize components of the extracellular matrix, cytoskeleton, and cell junctions at the VZ in PN2 littermates. We first investigated collagen IV (COL IV), a critical extracellular matrix protein that maintains the basement membrane in brain endothelial cells. One collagen IV subunit was downregulated in our RNA-Seq data (*Col4a6*), and another was downregulated and approaching statistical significance (*Col4a5*, see **Table 1** and **Supplementary File 1**). Our results in control pups show COL IV is highly expressed at the brain barriers, including in the choroid plexus (blood-cerebrospinal fluid barrier) and in the blood vessels of the VZ (**Figures 6A, B, D**, for colorblind compatible images see **Supplementary Figure 2**). Expression was also observed near the apical surface of the inferior VZ (**Figure 6B**). In contrast to euthyroid controls, PTU exposed pups show pronounced differences in the pattern of COL IV expression, specifically a notable reduction in the vasculature throughout the VZ (**Figures 6C, E** and **Supplementary Figures 3A–C**, see white arrows). Next, we visualized components of the cytoskeleton, given the RNA-Seq signal related to its formation and function. Filamentous actin (F-actin) was visualized by phalloidin staining in PN2 littermates. In euthyroid controls, F-actin is normally distributed throughout the brain, including in the VZ and its associated blood vessels (**Figures 6F, H** and **Supplementary Figure 3D**). In hypothyroid neonates, F-actin expression appeared globally reduced, and with a more punctate staining patterns as compared to controls (**Figures 6G, I** and **Supplementary Figure 3E**). This was apparent along the entire VZ (inferior, superior, and medial) and in the surrounding parenchyma (**Figures 6G, I** and **Supplementary Figures 3D, E**). This change in F-actin is reminiscent of the T4 and rT3-dependent change in actin polymerization, which has been reported in neurons and astrocytes *in vitro* and *ex vivo* by others (29–34). We next examined vimentin (VIM) immunostaining, another component of the cytoskeleton. Vimentin is an intermediate filament and used to visualize radial glial progenitor cells. Radial glia progenitors originate from neuroepithelial cells of the VZ, and anchor endfeet to this region (13). In our RNA-Seq dataset *Vim* was reduced in PTU exposed pups and this change was approaching statistical significance (q=0.06); *Pax6*, a transcription factor expressed by radial glia, was also downregulated (q=0.06, **Supplementary File 1**). Our results show that VIM is highly expressed in the PN2 control brain, with pronounced staining of radial glia cells as expected (**Figure 7A**). At high magnification, the spindle-like morphology of radial glial cells is clearly observed, including their attachment to the VZ (**Figure 7A**). In hypothyroid pups, VIM was still observed throughout the brain, although the apicobasal polarity of radial glial cells was abnormal (**Figure 7B**). This was clear at high magnification (**Figure 7B**).

As our data suggested that hypothyroid animals exhibited differences in the components that comprise cell junctions, we next asked whether adhesive proteins were also affected. Adherens junctions are the primary cell junction type of the VZ and are responsible for maintaining normal adhesion of the epithelium, including radial glia. This is supported by our results, where N-cadherin expression is strongly expressed in the VZ, especially along its apical border (**Figure 7C**, for colorblind compatible images see **Supplementary Figure 4**). In contrast, a loss of both basal and apical N-cadherin staining is observed in PTU exposed pups, including amongst the most luminal cells (**Figure 7D** and **Supplementary Figure 4D**, see arrows). In addition to adherens junctions, tight (occluding) junctions are crucial for polarization of epithelial cells, and for the normal functioning of the brain barriers. To visualize tight junctions, we examined the distribution of claudin 5 (CLD5). We did not observe any overt changes in the expression patterns of CLD5 in the brain parenchyma of the VZ, except along the apical surface; these changes were not as pronounced as N-cadherin (**Figures 7E, F**). CLD5 was also expressed in the CSF-facing epithelial cells of the choroid plexus (**Figure 7E**); interestingly, CLD5 expression appeared disorganized in the choroid plexus of PTU exposed neonates.

Next, as our bioinformatic analyses suggested that vascular patterning and/or function may be implicated in our RNA-Seq data, we analyzed the endothelial cell marker platelet endothelial cell adhesion molecule 1 (PECAM-1). PECAM-1 was observed in the vascular component of the choroid plexus, as well as in the brain’s blood vessels (**Figure 7E**). The PECAM-1 pattern at the VZ appeared less complex in PTU exposed pups as compared to controls, which was most apparent at the transition between the VZ-SVZ (**Figure 7F**). We did not observe any notable changes in the co-labeling of PECAM-1 and CLD5 between hypothyroid and euthyroid pups in the VZ parenchyma. However, the disorganization of normal CLD5/PECAM-1 expression was appreciable in the plexus of PTU exposed neonates (compare merge images of 7E and F, see C.P.). In total, these results showed differences in the expression patterns of extracellular matrix, cytoskeletal, and adhesive proteins within the posterior VZ. Surprisingly, during imaging we also observed clear differences in markers of the BBB (cerebral microvasculature, COL IV, PECAM-1, F-actin), as well as the blood-cerebrospinal fluid barrier (choroid plexus, COL IV, F-actin, PECAM-1, CLD5). It is noted that the choroid plexus data were gathered incidentally during imaging, and the RNA-Seq experiment did not include these cells.

### 3.5 TH action at the VZ may be mediated by both nongenomic and genomic mechanisms

**Figure 8:**
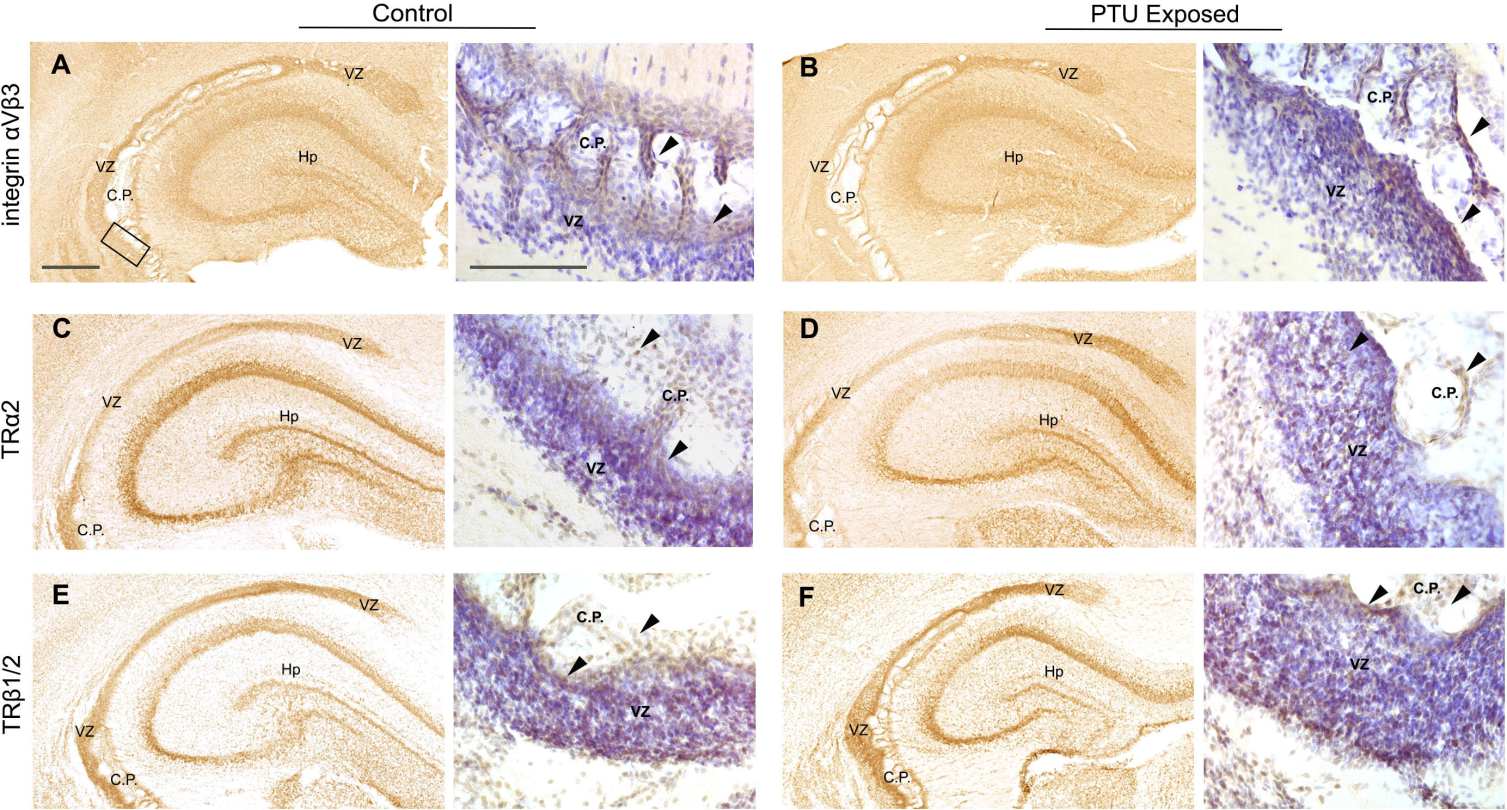
Mediators of TH action in PN2 pups. All receptors are visualized by diaminobenzidine (DAB) staining; at low magnification DAB is used alone and high magnification images were taken from sections counterstained with Cresyl violet. **(A)** Integrin α_V_β3 is expressed in the VZ of control and **(B)** PTU exposed pups. Signal was also detected in the hippocampus and choroid plexus. High magnification images of the inferior VZ (see bounding box in panel A) demonstrates enriched expression of integrin αVβ3 along the apical border of the VZ and in the choroid plexus (see arrows). **(C)** Thyroid receptor alpha 2 (TRα2) was detected in the VZ of both euthyroid control pups and **(D)** PTU exposed pups. High magnification images show TRα2 staining throughout the VZ and in the choroid plexus (arrows). **(E)** Thyroid receptor beta 1/2 (TRβ1/2) was also expressed in the VZ of both control and **(F)** PTU exposed pups, in similar brain compartments as integrin α_V_β3 and TRα2. At high magnification TRβ1/2 is also expressed in throughout the VZ and in choroid plexus cells (arrows). In all panels VZ, ventricular zone; C.P., choroid plexus; Hp, hippocampus; B.V., blood vessel; Vn, ventricle and scale bars are 50 µm. Low magnification images are stained with DAB alone, and high magnification images counterstained with Cresyl violet.

While we found that thyroid stimulating hormone receptor (*Tshr*) was differentially expressed in the posterior VZ (**Table 1**), the potential role of TSH in the VZ is unknown. However, it is expressed in the adult SVZ, suggesting it may be of importance in this region (35). No known receptor for T4 or T3 was identified as a DEG (**Supplementary File 1**). Given this lack of transcriptional signal, we chose to evaluate the localization of known T4/T3 receptors *in vivo*. Specifically, we hypothesized that the T4 receptor integrin αvβ3 would be highly expressed in the VZ. Integrins are transmembrane adhesion proteins that anchor intracellular F-actin to the extracellular matrix, serving as a cell-matrix junction (36). They are implicated in not only cell adhesion, but are critical to cell migration. Consistent with this hypothesis, we observed robust immunostaining of integrin αvβ3 in the VZ of both control and PTU exposed pups. (**Figures 8A, B**). We also detected its expression in the choroid plexus as well as in other brain compartments (**Figures 8A, B**). We next examined the expression of thyroid receptor isoforms, thyroid receptor alpha 2 and beta 1/2 (TRα2 and TRβ1/2). TRα2 and TRβ1/2 mediate genomic (nuclear initial action) and nongenomic (extranuclear initial action) TH signaling (see 37), and in addition to integrin αvβ3 in the VZ, could explain this region’s hormone sensitivity. Both *Thra* and *Thrb* were expressed in microdissected VZ cells although they were not differentially expressed between exposure groups; *Thra* exhibited higher expression as compared to *Thrb* (**Supplementary File 1**). Using an antibody that recognizes TRα2, the dominant negative isoform highly expressed in the developing rat brain (38), we showed that TRα2 is expressed in the VZ of both control and PTU exposed pups on PN2 (**Figures 8C, D**). TRα2 was also observed in the hippocampus, as well as in the choroid plexus (**Figures 8C, D**, Hp and C.P.). Next, using an antibody that recognizes TRβ1/2, robust expression was also observed in the VZ of both control and PTU exposed animals; the hippocampus, and choroid plexus also showed signal (**Figures 8E, F**). Strikingly, integrin αvβ3, TRα2, and TRβ1/2 show highly similar distribution in the PN2 telencephalon, including enrichment in the VZ (see **Supplementary Figure 5** for antibody control experiments). Taken together, this suggests that the VZ may be a “hotspot” of TH action in the developing brain.

## 4 Discussion

Neurodevelopment requires tightly coordinated spatiotemporal signaling processes, including those controlled by THs. Here we addressed the hypothesis that the posterior VZ is sensitive to TH action during the neonatal period in the rat. While the anterior and/or entire VZ/SVZ has been studied at different developmental stages including adulthood (39–44), the posterior VZ has not been investigated in isolation during an established time of TH dependency.

### 4.1 TH action affects components of cell junctions in the ventricular zone

Cell–cell junctions link cells to maintain tissue homeostasis, and regulate critical processes like tissue barrier function, cell proliferation, and cell migration. Tight and adherens junctions are two different multiprotein complexes that require similar components: adhesion proteins to physically connect cells, anchoring proteins that link the intercellular cytoskeleton, and an extracellular matrix that supports cell shape and contacts (45). Perturbation of any of these components can disrupt the structure and function of cell junctions. For example, disruption of CLD5 (adhesion molecule), actin (cytoskeleton), or COL IV (basement membrane, extracellular matrix) in tight junctions of brain endothelial cells will disturb the occluding activity of the BBB (46–48). In this study we show that various adhesion, cytoskeletal, and extracellular matrix proteins are simultaneously disturbed by moderate developmental hypothyroidism *in vivo*. While these findings may have several implications for the development and function of the brain, it is clear that cell migration is affected.

Our previous work to understand the cellular etiology of the periventricular heterotopia showed that radial glia cells were abnormal in hypothyroid rat pups (4). We correlated this observation to downregulation of *Spred1*, as detected by qRT-PCR in a hand-dissected region of the brain that included the cortex, hippocampus, and VZ (2, 4). Here, using a lower dose of PTU and laser capture microdissection to isolate the VZ in newborn rats, we again demonstrate downregulation of *Spred1* and loss of radial glia apicobasal polarity. Phoenix and Temple (49) showed that *Spred1* knockdown in the embryonic mouse brain caused reduced cell adhesion at the VZ/SVZ, abnormal morphology of radial glia cells, and disordered neuroblast migration. The authors also detected periventricular heterotopia in the posterior telencephalon of postnatal mice (49). In patients, periventricular heterotopia are often attributed to mutations in cytoskeletal proteins. The most commonly associated are within filamin A (FLNA), but other genes encoding cytoskeletal, adhesive, and extracellular matrix proteins are also implicated (reviewed in 50). Intriguingly, various COL IV mutations cause not only heterotopia, but also small vessel disease of the brain in affected individuals (50). Here we also show that TH action affects expression of *Col4a6* (q*=0.04)*, *Col4a5* (q*=0.07)*, and COL IV at the rat VZ. Regardless of the molecular etiology, it is established across species that heterotopia are caused by abnormalities in radial glia-mediated cell migration (49–52). These observations, coupled with our repeatable findings, strengthens the interpretation that TH action affects radial glia form and function, which then leads to heterotopia formation in the rat.

The conclusion that radial glial cells and/or cell migration can be affected by TH action has been supported by others (8, 53–60). There are several hypotheses about why this occurs, including TH-mediated loss of cell adhesion at the VZ, which normally anchors radial glia endfeet (4, 59). Other hypotheses include direct changes in the cytoskeleton of radial glia, as F-actin in particular is responsive to T4 and rT3 in the brain (29–34, 61). Given the data presented here, we cannot determine whether abnormalities in adherens junctions of the VZ cause loss of radial glial cell morphology, or if this is a secondary effect of cytoskeletal changes in radial glia themselves. Regardless, loss of adherens junctions in the VZ is associated with loss of radial glia apicobasal polarity, which results in abnormal cell migration in other models (62). Interestingly, despite the VZ’s function as a stem cell niche, we did not detect a robust transcriptional signal related to neurogenesis and/or cell differentiation. For example, we did not detect a downregulation of genes related to oligodendrocyte progenitors (i.e., *Ng2*, *Olig1*, *Olig2*) in PTU exposed animals, even though reduced myelination is well established consequence of hypothyroidism (1). We acknowledge that bulk RNA-Seq may make such determinations regarding cell population changes difficult (63). But overall, the RNA-Seq data are supportive of our previous work, where we did not find a significant difference in Ki67+ cells at the ventricular epithelium in PN2 hypothyroid neonates (4). Neurogenesis in the developing and adult VZ-SVZ has been shown to be affected by THs (39, 42, 44, 64, 65), but the majority of published work has examined the anterior or entire VZ and/or SVZ in rodents older than this study. Some cellular biology studies have shown that abnormal cell junctions in the brain precede neurogenesis and cellular differentiation deficits, due to a breakdown in intercellular communication (reviewed in 66). We also show by pathway analysis that oxidative phosphorylation was significantly represented in our RNA-Seq data, and that it was estimated to be activated. Neural progenitor cells switch primarily from glycolysis to oxidative phosphorylation upon differentiation (67), so it is possible that this transcriptional signal may be another early indicator of abnormal neurogenesis in hypothyroid animals. In the future it would be interesting to determine if and how TH-action may affect cell differentiation in this model of moderate hypothyroidism, but developmental stages later than PN2 should be included.

TH action may influence the cytoskeleton and extracellular matrix of brain tissue by multiple mechanisms (29, 31–33, 61). With respects to *in vivo* models, Morte et al. reported differential expression of cytoskeletal genes in the brains of hypothyroid rat pups; however, maternal TH function was abolished by thyroidectomy as well as methamizole exposure 68. In a series of *ex vivo* experiments, Farwell et al. showed that deficiency in T4 and rT3 reduced F-actin in rat cerebellar cells, which attenuated neurite migration (33). This migration phenotype could be recapitulated by blocking the integrin β1 subunit, even in the presence of sufficient T4/rT3 concentrations. This suggests that that the hormonal regulation of F-actin is necessary for normal integrin β1-mediated cell migration (33). With respects to this study, brain T4 was significantly reduced although rT3 was more variable between rat pups across litters. Integrin αvβ3 protein was also expressed in the developing rat VZ. Similar to *in vitro* and *ex vivo* reports, we demonstrated that F-actin appeared reduced and our model suffers from abnormal cell migration as evidenced by periventricular heterotopia formation (3, 5–8). Thus, the similarities between experimental models are compelling and suggests the brain cytoskeleton can be perturbed by moderate hypothyroidism *in vivo*. In the future, additional studies are needed to fully elucidate the TH signaling mechanism(s) at the VZ and how this translates to the observed cellular abnormalities. This could include rescue experiments where T4/T3 are each supplemented to hypothyroid pups and gene expression measured at the VZ, chromatin immunoprecipitation of thyroid receptors, and/or pharmacological manipulation of integrin αvβ3.

### 4.2 TH action and the blood-brain and blood-cerebrospinal fluid barriers

We previously hypothesized that the VZ was susceptible to TH action, due to its juxtaposition between two sources of brain THs: the CSF and an enriched vascular network (4). The VZ itself also represents the developmentally transient CSF-brain barrier (69, 70). What we did not expect to discover was that the TH-dependent changes in the VZ parenchyma may also extended to cerebral endothelial cells.

The blood-brain and blood-CSF barriers develop in parallel with the brain tissue, and these fluid interfaces are functional during fetal development (71, 72). These mechanical and physiological barriers are comprised of tight and adherens junctions that line endothelial cells of the central nervous system, expression and activity of influx and efflux transporters, and fluid flux-flow dynamics (73). However, tight junctions are the hallmark of their occluding activity. In our RNA-Seq experiment, we sequenced all cell types within microdissected VZ, which represents a heterogeneous population. We subsequently identified several DEGs that are critical to brain barrier function (**Table 1**); microscopy also revealed pronounced effects in endothelial cells of the brain vasculature and/or choroid plexus, in addition to changes in VZ parenchyma. Specifically, COL IV, F-actin, CLD5, and PECAM-1 are associated with endothelial cell patterning and/or function of the BBB *in vivo* (46, 74, 75), and we discovered clear effects in their localization between euthyroid and hypothyroid neonates. These preliminary findings pose the new question – could brain barrier development and/or function be an underappreciated target of TH action?

Several studies in adult and canines demonstrate that hypothyroidism is associated with increased permeability of the brain barriers (76–81). Clinically, this manifests as increased protein concentrations in the CSF. In one study, necropsy of affected hypothyroid dogs suggested cerebrovascular disease in this species (80). More than half a century ago, Thompson et al. studied 17 adult patients with myxedema, who also presented with increased CSF protein concentrations (76). Amazingly, after supplementation with thyroid extract all but 2 patients had a marked reduction in CSF protein content, and achieved normal levels once euthyroidism was established (76). This rescue to a normal phenotype demonstrates that brain barrier function directly responds to TH action. While the mechanisms of these observations are unknown, TH signaling can affect vascular function and patterning in different tissues. This has been shown *in vitro* and *in vivo* with regards to T4/rT3 action on integrin αvβ3 (reviewed in Davis et al., 2020), as well as TSH activity *via* TSHR in primary human cultures (82). Both pathways appear to be proangiogenic, where both excess T4 or TSH can induce angiogenesis (36, 82). It has also been shown in rats exposed to PTU from birth to PN21 that brain angiogenesis was reduced, including in the complexity and density of microvessels (83). Taken together, there is evidence that THs control the morphogenesis and function of endothelial cells, and this is likely mediated by multiple complex mechanisms. While there is convincing evidence that thyroid-mediated brain barrier disruption in adults is transient and corrects following TH supplementation, this may not occur during development. If TH action controls patterning of cells that comprise the brain barriers, then it is possible that developmental hypothyroidism could lead to permanent changes in the way the barriers are formed and/or function. Future studies should determine if TH action can affect brain barrier activity, and if so, determine its persistence. There is accumulating evidence that both neurodevelopmental and neurodegenerative disorders are associated with increased permeability of the BBB (84), so any TH-mediated effects could have significant consequences.

### 4.3 The human health implications of this study

This study extends our previous observations and reinforces that TH action targets multiple components of normal cell junctions in the developing brain. These abnormalities can converge to affect downstream processes like cell migration and potentially brain barrier function. While previous *in vivo* work has demonstrated that radial glia morphology and/or cell migration is affected by developmental hypothyroidism, these conclusions were drawn from experiments that induced severe TH disturbances (e.g., maternal thyroidectomy and/or high doses of TH modulators) (53–57, 59, 60). While these studies are critical to our understanding of mechanisms, it can be difficult to translate these findings to patients. Our PTU exposed dams exhibited a relatively minor 72% increase in TSH, with no significant change in serum T3. The American Thyroid Association advises that normal TSH in pregnant patients during the second trimester is 0.2-3.0 mIU/L in absence of a laboratory-established reference range (85). Given the biological variability of TSH in human populations and our presented data, we consider our experiment representative of moderate maternal hypothyroidism. The pronounced effects we observed at the neonatal VZ, with the knowledge that a 3 ppm PTU exposure will induce a periventricular heterotopia in offspring (3, 5, 6), emphasizes that euthyroidism throughout pregnancy is critical. The mammalian brain undergoes protracted development, so processes like cell migration occur over many weeks in both human and rodents (13, 86).

In conclusion, this study provides evidence that the posterior VZ is sensitive to THs in newborn rats, even under conditions of moderate maternal hypothyroidism. Specifically, components of normal cell junctions including adhesive, cytoskeletal, and extracellular matrix transcripts were differentially expressed as detected by RNA-Seq and pathway analyses. Immunofluorescence reinforced these results, and supports that cell migration is a target of TH action. In addition, this work led to the unexpected finding that components of both the blood-brain and blood-cerebrospinal fluid barrier may also be affected by hypothyroidism. While we acknowledge our study’s limitations, namely that there are multiple cell types in this the developing VZ and we performed bulk RNA-Seq, these findings support previous hypotheses that cell adhesion and radial glia cell polarity are affected in this critical region. In the future, single cell RNA-Seq to differentiate between various cell types in the VZ (i.e., radial glia versus endothelia cells), and functional studies of brain barrier function could further illuminate mechanisms of TH action.

## Data availability statement

All raw data for hormone analyses can be found in the public repository Science Hub. The sequencing data discussed in this publication have been deposited in NCBI's Gene Expression Omnibus (87) and are accessible through GEO Series accession number GSE218930 (https://www.ncbi.nlm.nih.gov/geo/query/acc.cgi?acc=GSE218930).

## Ethics statement

All experiments were conducted with prior approval from the United States Environmental Protection Agency’s (US EPA’s) Institutional Animal Care and Usage Committee and were carried out in an Association for Assessment and Accreditation of Laboratory Animal Care approved facility.

## Author contributions

KO conceived and executed the study, performed sectioning, laser capture microdissection, library preparations, bioinformatics, microscopy, prepared figures, and wrote the article. AS performed sectioning and immunohistochemistry. BM assisted with bioinformatics. KB and MG assisted with the animal exposure. CR, JF, and RG measured thyroid hormones. TS provided equipment, and AP also provided equipment and training for laser capture microdissection. All authors contributed to the article and approved the submitted version.
