# Supplementary figures and images for: Thyroid hormone action controls multiple components of cell junctions at the ventricular zone in the newborn rat brain
Source: Front Endocrinol (Lausanne). 2023 Feb 10;14:1090081. doi: 10.3389/fendo.2023.1090081 (PMC9950412; doi:10.3389/fendo.2023.1090081)

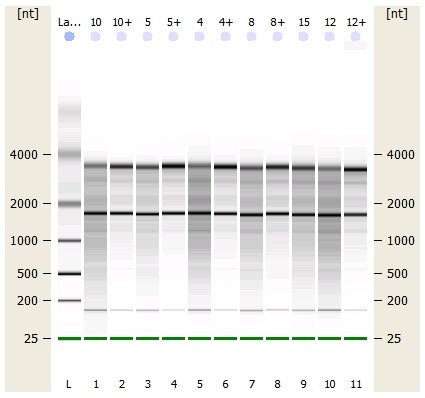

Supplement: Supplementary file 1 [file Image_1.jpg]

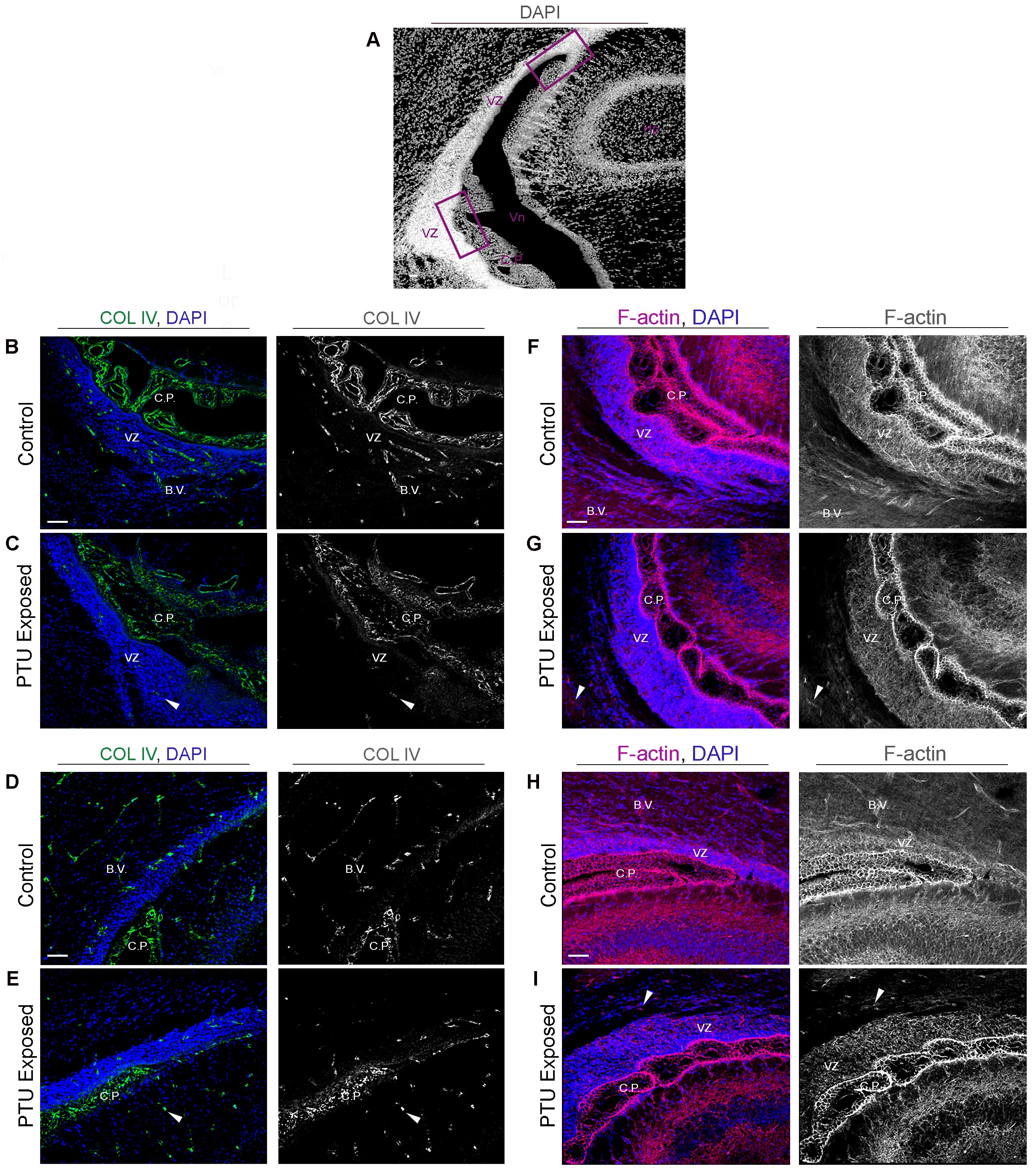

Supplement: Supplementary file 2 [file Image_2.jpg]

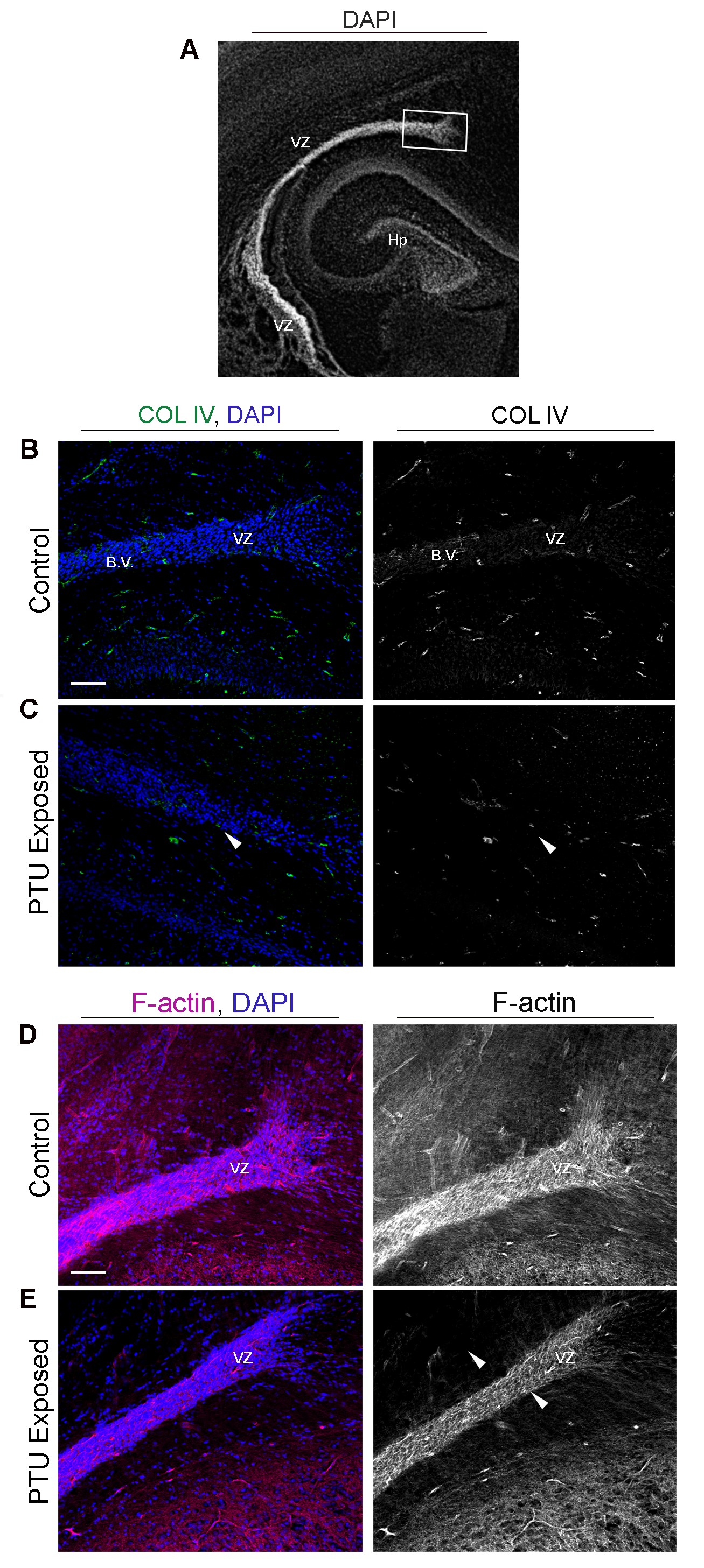

Supplement: Supplementary file 3 [file Image_3.jpeg]

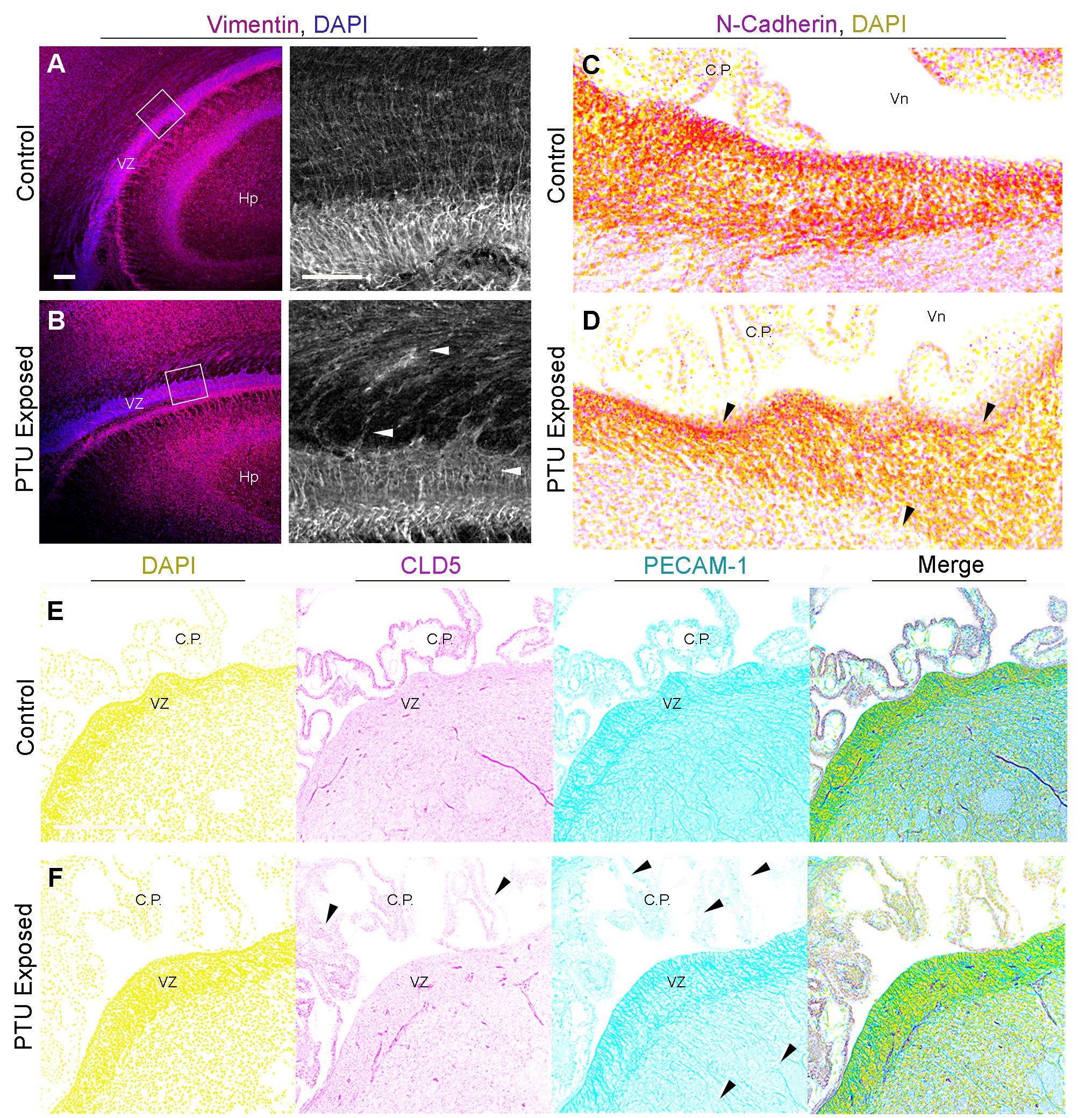

Supplement: Supplementary file 4 [file Image_4.jpg]

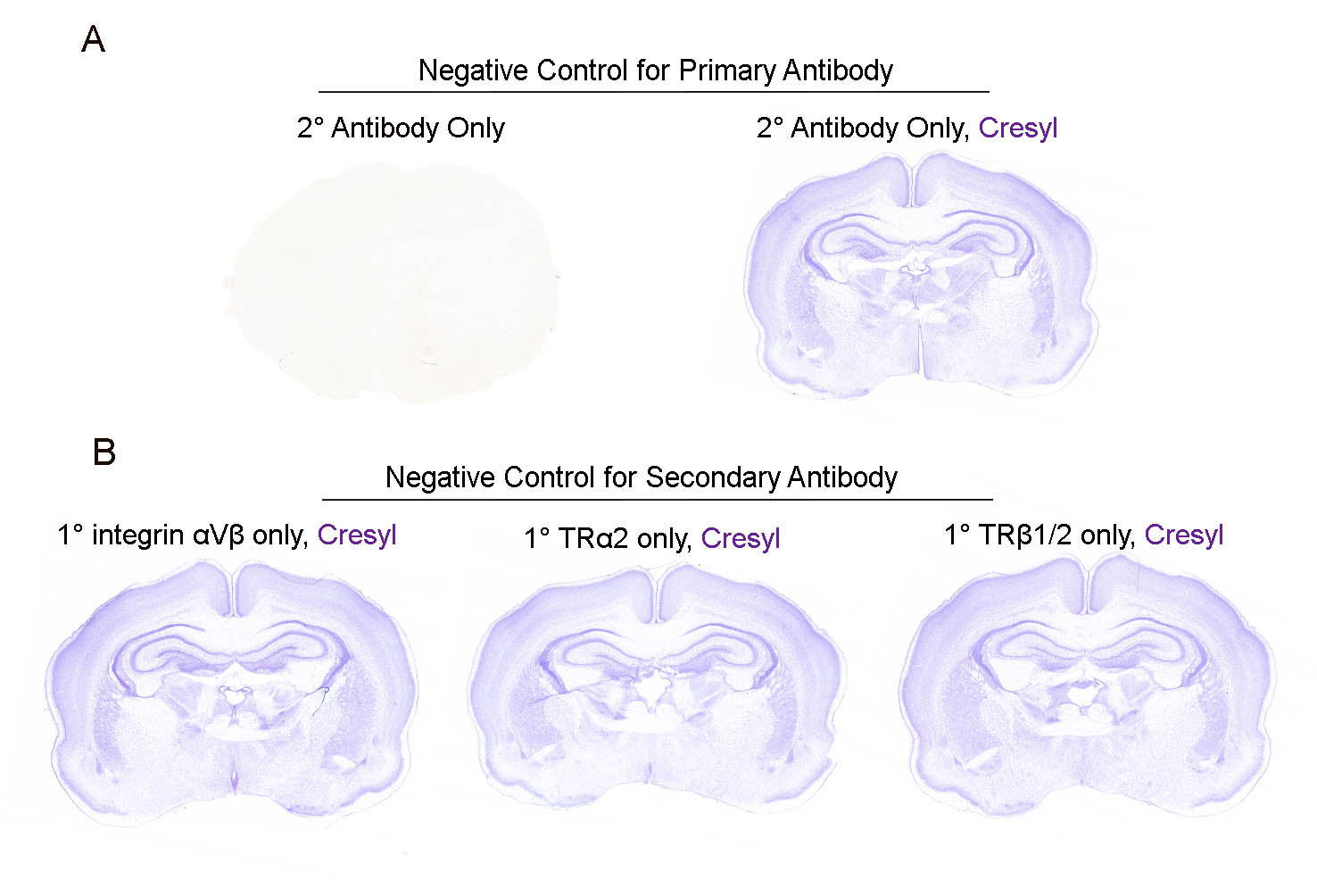

Supplement: Supplementary file 5 [file Image_5.jpg]
