# Supplementary material for: Thyroid hormone action controls multiple components of cell junctions at the ventricular zone in the newborn rat brain
Source: Front Endocrinol (Lausanne). 2023 Feb 10;14:1090081. doi: 10.3389/fendo.2023.1090081 (PMC9950412; doi:10.3389/fendo.2023.1090081)
Supplement: Supplementary file 10 [file Table_1.docx]

| Primary Antibody | Concentration | Secondary Antibody | Concentration | Laser Line |
| --- | --- | --- | --- | --- |
| Sox2  Abcam, ab97959 | 1:250 | Abcam, ab150083 | 1:500 | 488 |
| Collagen IV  Abcam, ab236640 | 1:100 | Abcam, ab150083 | 1:500 | 488 |
| Vimentin  Abcam, ab24525 | 1:300 | Abcam, ab150175 | 1:500 | 638 |
| N-Cadherin  Santa Cruz, sc-59987 | 1:200 | Abcam, ab150117 | 1:500 | 561 |
| Claudin-5  Thermofisher, 34-1600 | 1:100 | Abcam, ab150083 | 1:500 | 488 |
| PECAM-1  Santa Cruz, sc-376764 | 1:50 | Abcam, ab175701 | 1:500 | 561 |
| Integrin αVβ3  Thermofisher Scientific, BS-1310R | 1:200 | Biotinylated and ABC, Vector PK-4001 and PK-4002 | Per manufacturer’s directions | N/A |
| TRα2  Novus, NBP1-90118 | 1:500 | Biotinylated and ABC, Vector PK-4001 and PK-4002 | Per manufacturer’s directions | N/A |
| TRβ1/2  Abcam, ab5622 | 1:500 | Biotinylated and ABC, Vector PK-4001 and PK-4002 | Per manufacturer’s directions | N/A |

**Supplementary Table 1. Antibodies used in this study.**
